# The Basis for Targeting the Tumor Macrophage Compartment in Glioblastoma Immunotherapy

**DOI:** 10.3390/cancers17101631

**Published:** 2025-05-12

**Authors:** Thomas Eckert, Chase Walton, Marcus Bell, Coulter Small, Nathan C. Rowland, Charlotte Rivers, Alicia Zukas, Scott Lindhorst, Peter Fecci, Ben A. Strickland

**Affiliations:** 1 School of Medicine, University of South Carolina, Columbia, SC 29209, USA; 2 MUSC Institute for Neuroscience Discovery, Charleston, SC 29425, USA; rowlandn@musc.edu; 3 College of Medicine, Medical University of South Carolina, Charleston, SC 29425, USA; waltonch@musc.edu (C.W.); bellmar@musc.edu (M.B.); 4 Department of Neurosurgery, Medical University of South Carolina, Charleston, SC 29425, USA; smallcou@musc.edu (C.S.); zukas@musc.edu (A.Z.); lindhors@musc.edu (S.L.); strickbe@musc.edu (B.A.S.); 5 Department of Radiation Oncology, Medical University of South Carolina, Charleston, SC 29425, USA; iveyc@musc.edu; 6 Hollings Cancer Center, Medical University of South Carolina, Charleston, SC 29425, USA; 7 Department of Neurosurgery, Duke University, Durham, NC 27710, USA; peter.fecci@duke.edu

**Keywords:** glioblastoma, immunotherapy, tumor microenvironment, tumor-associated macrophages, macrophage polarization

## Abstract

**Background:** Glioblastoma (GBM) remains the most aggressive primary brain tumor with limited treatment options. The immunosuppressive tumor microenvironment (TME), largely shaped by tumor-associated macrophages (TAMs), represents a significant barrier to effective immunotherapy. **Objective:** This review aims to explore the role of TAMs within the TME, highlighting the phenotypic plasticity, interactions with tumor cells, and potential therapeutic targets to enhance anti-tumor immunity. **Findings:** TAMs constitute a substantial portion of the TME, displaying functional plasticity between immunosuppressive and pro-inflammatory phenotypes. Strategies targeting TAMs include depletion, reprogramming, and inhibition of pro-tumor signaling pathways. Preclinical studies show that modifying TAM behavior can shift the TME towards a pro-inflammatory state, enhancing antitumor immune responses. Clinical trials investigating inhibitors of TAM recruitment, polarization, and downstream signaling pathways reveal promising yet limited results, necessitating further research to optimize approaches. **Conclusions:** Therapeutic strategics targeting TAM plasticity through selective depletion, phenotypic reprogramming, or modulation of downstream immunosuppressive signals represent promising avenues to overcome GBM-associated immunosuppression. Early clinical trials underscore their safety and feasibility, yet achieving meaningful clinical efficacy requires deeper mechanistic understanding and combinatorial approaches integrating macrophage-direct therapies with existing immunotherapeutic modalities.

## Simple Summary

This review highlights the role of tumor-associated macrophages (TAMs) in shaping the immunosuppressive tumor microenvironment (TME) of glioblastoma (GBM), an aggressive brain tumor with limited treatment options. TAMs, which constitute a substantial portion of the TME, exhibit high phenotypic plasticity and dynamically shift between pro-inflammatory and immunosuppressive states. GBM exploits this plasticity to drive tumor growth by inducing TAM polarization toward the immunosuppressive phenotype through mechanisms involving cytokine secretion, immune checkpoint pathways, and metabolic interactions. This polarization contributes to immune evasion, tumor proliferation, angiogenesis, and treatment resistance. Therapeutic strategies targeting TAMs include depletion, reprogramming toward the pro-inflammatory phenotype, and inhibiting pro-tumor signals. Several approaches are under investigation, such as blocking CSF-1R, disrupting CCL2/CCR2 and PI3K pathways, targeting PD-L1 expression, and utilizing nanoparticle-based delivery systems for selective TAM modulation. Further research targeting TAM plasticity and polarization is essential for developing treatments that overcome GBM’s robust immunosuppressive TME and improve patient survival.

## 1. Introduction

Glioblastoma (GBM) is the most common primary malignant brain tumor with an annual incidence in the U.S. of three cases per 100,000 persons [1]. Despite conventional treatment consisting of surgery and chemoradiotherapy, GBM remains a grim diagnosis with a median survival time of 15 months [2,3]. The current gold standard only modestly extends survival and thus, there is an urgent need for novel treatment strategies. Although immunotherapy has demonstrated efficacy for both solid and blood cancers [4,5,6], it has thus far failed to improve survival in GBM [7,8,9]. There are several potential contributing obstacles to immunotherapy targeting the adaptive immune response (i.e., checkpoint inhibition, oncolytic viral therapy, and vaccine-based therapy) which can be simplified to a paucity of T cells and persistent T cell exhaustion [10]. Given that GBM is characterized by a “cold” tumor microenvironment, a promising strategy is to counteract tumor-derived immunosuppressive factors that exhaust and deplete T lymphocytes while impairing antigen presentation [11,12,13]. This necessitates the advancement of immunotherapeutic strategies targeting non-T cell immune cell populations to counteract drivers of the immunosuppressive GBM tumor microenvironment (TME). 

Tumor-associated macrophages (TAMs) represent a substantial component of the TME, composing roughly 30% at initial diagnosis and up to 60% at tumor recurrence [11,14] These macrophages exhibit diverse classifications based on their origin, surface markers, and functions [15,16]. Initial investigations using immunohistochemistry and flow cytometry techniques once categorized TAMs into a near binary state—labeling immunosuppressive (protumor) cells as “M2-like” and pro-inflammatory (antitumor) cells as “M1-like” [15]. However, with the advent and implementation of more sophisticated scientific methodology, such as transcriptomics, we now realize the TAM compartment displays a high degree of cellular plasticity and is capable of a shifting continuum of phenotypes based on external and internal factors [15]. This malleable and populous immune cell compartment therefore represents a potential target for therapeutic opportunities by shifting the overall functioning towards antitumor behavior. 

## 2. Nomenclature

The original M1/M2 classification subclassifies macrophages based on their fundamental role in immune response but has since expanded due to advancements in single cell analyses and understanding of glioma cell heterogeneity [15]. In recent years, M1/M2 binary classification has been challenged as macrophage polarization has been revealed to be a dynamic and shifting continuum from pro-inflammatory to immunosuppressive phenotypes, respectively [15]. In GBM, TAMs exhibit functional plasticity, primarily polarizing into one of the two phenotypes [15]. The pro-inflammatory phenotype (classically activated macrophages, “M1”) acts to suppress GBM progression, while the immunosuppressive phenotype (alternatively activated, “M2”) supports tumor growth [15]. The activation of pro-inflammatory TAMs occurs through multiple signals, including interferon gamma (IFN-g), granulocyte-macrophage colony stimulating factor (GM-CSF), lipopolysaccharide (LPS), and tumor necrosis factor-alpha (TNF-a). Once activated, they subsequently also secrete pro-inflammatory cytokines such as TNF-α, IL-1β, IL-6, IL-8, IL-12, and IL-23, which work together to suppress tumor growth [15,17,18,19]. However, as tumors progress, GBM can induce the repolarization of TAMs into the immunosuppressive phenotype. This repolarization is typically mediated through different pathways and interleukins, primarily involving peroxisome proliferator-activated receptor-γ (PPAR γ), STAT6, IL-4, IL-13, TGF-β, and colony-stimulating factor (CSF)-1 [15,17,18,19]. Repolarized TAMs contribute to an immunosuppressive extracellular milieu maintained by secreted factors including arginase 1, IL-13, IL-10, IL-4, vascular endothelial growth factor (VEGF), TGF-β, CCL22, and CCL17 [15,17,18,19]. Secretion of these factors promotes tumor progression in multiple ways, including stimulating angiogenesis, facilitating immune cell infiltration, assisting in tissue remodeling, and contributing to drug resistance. Additionally, immunosuppressive TAMs enhance the Th2 immune response to further promote tumor growth and prevent anti-tumor activity within the TME [17,18].

**Figure 1:**
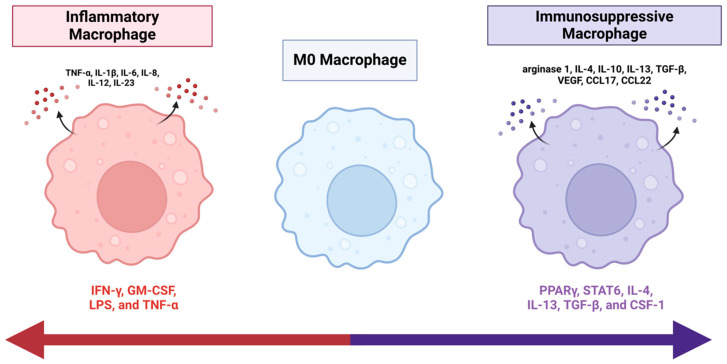
Polarization Continuum. M0, naïve macrophages are induced by various activating factors to polarize into pro-inflammatory or immunosuppressive phenotypes. Pro-inflammatory macrophages (left) are induced by IFN-γ, GM-CSF, LPS, and TNF-α and release pro-inflammatory cytokines such as TNF-α, IL-1β, IL-6, IL-8, IL-12, and IL-23. Immunosuppressive macrophages (right) are activated by PPARγ, STAT6, IL-4, IL-13, TGF-β, and CSF-1 and release anti-inflammatory mediators including arginase 1, IL-4, IL-10, IL-13, TGF-β, VEGF, CCL17, and CCL22. Created in BioRender.

Studies using single-cell RNA sequencing (scRNA-seq) and advanced proteomic techniques have shown that TAMs frequently co-express markers associated with both pro-inflammatory and immunosuppressive phenotypes, such as IL-10 and TNF-α, or CD86 and CD206, suggesting remarkable plasticity [15]. This complexity is influenced by factors including tumor mutational status, molecular subtype, and treatment conditions, with macrophages in *IDH* wild-type gliomas showing a tendency toward immunosuppressive phenotypes driven by cytokines like midkine, ANXA1, and GPNMB [15]. Moreover, mesenchymal GBM subtypes, which express elevated levels of both immunosuppressive and pro-inflammatory genes, appear particularly reliant on TAMs to reinforce their aggressive phenotype [15]. Targeting TAMs and their respective pathways has emerged as a novel treatment modality to shift the continuum in favor of the pro-inflammatory phenotype (Figure 1). 

## 3. Barriers to Immunotherapy

The tumor microenvironment (TME) consists of an intricate network of blood and lymphatic vessels, extracellular matrix (ECM), immune and tumor cell populations, and an extracellular, immunosuppressive signaling milieu that contributes to tumor growth [12,17]. The TME is rich in immunomodulatory cytokines, including transforming growth factor-beta (TGF-β), interleukin-10 (IL-10), and prostaglandin E2 (PGE2). These promote an immunosuppressive state by shifting brain-resident microglia, and infiltrating TAMs, toward the pro-tumor phenotypes that support tumor growth and downregulate anti-tumor immune responses [20]. Additionally, GBM cells frequently express checkpoint molecules like PD-L1 and CTLA-4 which contribute to T-cell exhaustion and immune evasion [20]. Additional immunosuppressive factors within the TME, including indoleamine 2,3-dioxygenase (IDO) and interleukin-6 (IL-6), recruit myeloid cells and promote lymphocyte differentiation into regulatory T cells (Tregs), leading to dampened anti-tumor T-cell responses [21]. Furthermore, IDO modulates immune responses by catabolizing tryptophan into kynurenine, leading to further expansion of Tregs and suppression of effector T cell function. IDO also facilitates the recruitment and activation of circulating Tregs within the tumor microenvironment. IL-10 acts as a potent anti-inflammatory cytokine by sustaining FoxP3 expression on Tregs and enhancing PD-L1 expression on TAMs and peripheral monocytes, contributing to decreased activation of cytotoxic T lymphocytes [21]. The combined actions of IL-10 and IDO synergistically downregulate anti-tumor immunity in GBM, enabling immune evasion and promoting tumor progression [21]. 

In addition to its cellular and molecular barriers, GBM is further protected by the structural and functional complexity of the blood–brain barrier (BBB). Comprised of non-fenestrated endothelial cells with adherens and tight junction proteins, the BBB restricts the entry of hydrophilic drugs, biologics, and lipophilic agents via active efflux mechanisms, thus limiting brain drug accumulation [22]. While the BBB is partially disrupted in GBM, it often remains intact in peritumoral regions, contributing to heterogenous drug delivery and protecting infiltrative tumor cells from chemo- and immunotherapies [23]. Although the CNS was historically viewed as immunoprivileged, recent evidence of functional lymphatics and T-cell trafficking indicates immunotherapeutic strategies are viable [23].

Despite GBM’s localized growth, patients frequently exhibit systemic immunosuppression, marked by decreased T cell counts and reduced lymphoid organ sizes, akin to immunodeficiency conditions [24]. This contributes to the systemic sequestration of naïve T cells in the bone marrow, linked to the loss of sphingosine-1-phosphate receptor 1 (S1PR1) on T cells which impairs their egress from lymphoid organs [24]. This systemic immunosuppression occurs in treated and untreated patients alike, indicating that it is intrinsic to the disease rather than a consequence of chemotherapy or corticosteroids [24]. This immunosuppression is often exacerbated by GBM standard-of-care chemotherapy. 

The multiple mechanisms of immune cell dysfunction in the TME drive a reduced T cell population consisting of exhausted and anergic effector lymphocytes [10]. The role of TAMs in this “T cell desert” has been of recent interest. Chronic antigen exposure via TAMs leads to the exhaustion of both progenitor and terminally differentiated T cell populations [25,26]. This process, in part, is mediated by MHC class I antigen presentation on the cell surface of TAMs [25]. While therapeutic TAM depletion may increase cytotoxic T lymphocyte number and anti-tumor activity, here, we will argue that macrophage reprogramming is a superior strategy given the unique role of TAMs in shaping immune function within the TME [25,27]. 

## 4. GBM–TAM Interactions

The interaction between GBM cells and TAMs involves multiple signaling factors which serve two primary functions: recruiting TAMs to the TME and promoting their polarization toward the pro-tumor M2 phenotype. Through this mechanism, GBM cells effectively reprogram TAMs to support tumor persistence [28]. GBM-secreted cytokines remodel the TME and also act as potent chemo attractants. These include C-X3-C motif chemokine ligand 1 (CX3CL1), C-C motif chemokine ligand 2 (CCL2), macrophage inhibitory cytokine-1 (MIC-1), and colony-stimulating factor 1 (CSF1) [28]. The recruitment of TAMs to the glioblastoma tumor microenvironment is primarily mediated by CX3CL1, which binds to CX3C receptor 1 (CX3CR1) expressed on peripheral macrophages to promote their residency within the TME. Additionally, glioblastoma cells secrete C-X-C motif chemokine ligand 16 (CXCL16), which influences both TAMs and tumor cells through its receptor C-X-C motif chemokine receptor 6 (CXCR6). In TAMs, CXCL16/CXCR6 signaling drives polarization toward the immunosuppressive phenotype. This signaling axis also directly enhances GBM cell proliferation, migration, and invasion, as demonstrated both in in vitro and in mouse glioma models [28]. Current therapeutic strategies targeting TAMs focus on depletion, reprogramming, or mitigating downstream protumor effects to rebalance the TME towards a more antitumor phenotype [29,30]. 

## 5. Depleting the TAM Population

### 5.1. Prevent Trafficking into the TME

**Figure 2:**
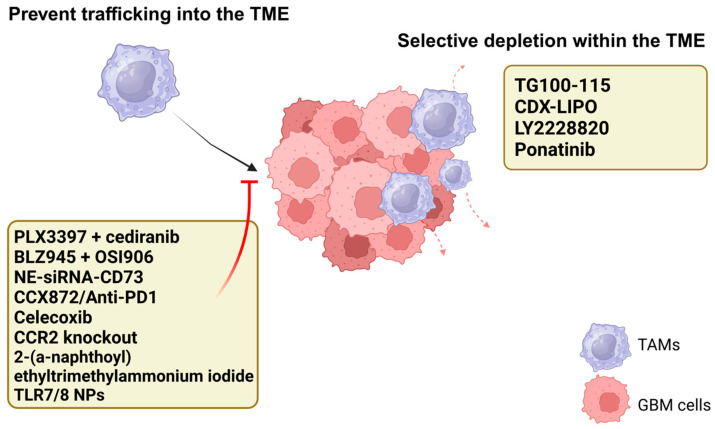
Various therapeutic strategies to prevent TAM trafficking into the TME or selectively deplete from within include CSF-1R inhibition via PLX3397 or BLZ945, CCL2/CCR2 axis disruption via CCX872 or CCR2 knockout, and PI3K pathway inhibition via TG100-115 or CDX LIPO.

Multiple strategies have been employed to prevent TAM trafficking into the TME or selective depletion of TAMs within the TME (Figure 2). The signaling axis of colony-stimulating factor 1 (CSF-1) and its receptor, CSF-1R, serves as a fundamental regulator in the development and maintenance of both microglia and macrophages. This pathway plays an essential role in governing the survival, proliferation, and differentiation of these myeloid cell populations, thereby maintaining their homeostatic functions within the tissue microenvironment [30,31]. Resistance to CSF-1R inhibitors is likely mediated through the interaction of insulin-like growth factor-1 (IGF-1) and tumor cell IGF-1 receptor (IGF-1R), causing an upregulation of the phosphoinositide 3-kinase (PI3K) pathway [32]. The binding of IGF-1 to IGF-1R triggers PI3K pathway upregulation in tumor cells. Notably, concurrent inhibition of IGF-1/PI3K and CSF-1R using Linsitinib (OSI906) and Sotuletinib (BLZ945) has shown promising results, significantly extending median survival in preclinical models until tumor recurrence [32]. The efficacy of CSF-1R inhibition appears to be influenced by tumor molecular subtypes. Single-cell RNA sequencing has demonstrated that PDGFB-driven pro-neural tumors, characterized by microglial dominance, respond favorably to PLX3397 treatment through downregulation of pro-tumor gene expression [33]. In contrast, RAS-driven mesenchymal-like tumors demonstrate resistance to PLX3397 monotherapy or combination therapy with anti-PI3K pathway agents, attributed to pro-inflammatory and pro-angiogenic signaling in their TAM populations [34]. Co-targeting of TAMs and VEGF with the combination PLX3397 and cediranib (AZD2171), a VEGF-inhibitor, revealed decreased angiogenesis and vessel density as well as reduced tumor cell proliferation in RAS-driven mesenchymal GBM [33].

Adenosine production, regulated by CD73, is another mechanism by which GBM suppresses pro-inflammatory cytokine reduction, decreases effector T cell response, and induces immunosuppressive macrophage polarization. Using a cationic nanoemulsion siRNA CD73 formulation, researchers assessed tumor cell apoptosis, immune modulation, and cytokine profiles in GBM-bearing rats [34]. The treatment induced significant apoptosis, reduced tumor volume by 60–70%, and selectively decreased CD73 expression in GBM cells and TAMs. Immunophenotyping revealed reduced populations of Tregs, microglia and TAMs in the TME, with decreased immunosuppressive polarization markers. Pro-inflammatory markers IL-6, CCL17, and CCL22 increased post-treatment; thus, this study suggests that limiting adenosine-mediated immunosuppression can modulate the TME by reducing the population of immunosuppressive TAMs while simultaneously promoting a shift to a pro-inflammatory environment [34]. 

The complement receptor 2 and C-C motif chemokine ligand 2 (CCR2/CCL2) axis represents another potential target as it plays a role in recruitment of Tregs and Myeloid-Derived Suppressor Cells (MDSCs) that promote immunosuppression in the TME [35]. An antagonist of CCR2, CCX872, increased survival outcomes in preclinical models as a monotherapy and in combination with immune checkpoint inhibition of PD-1 [36]. CCX872 led to a reduction in MDSCs and an increase in effector T lymphocytes at the TME, as well as decreased markers of T cell exhaustion with combination therapy [36], establishing a potential avenue to pursue in early human trials. The nonsteroidal anti-inflammatory drug, celecoxib, has shown ability to induce apoptosis of tumor cells by decreasing the expression of CCL2 and CXC motif chemokine ligand 10 (CXCL10), further demonstrating the potential this pathway must influence downstream immunosuppression and tumor cell persistence [37]. 

The mesenchymal subtype of GBM is associated with increased chemoresistance and poorer survival outcomes, partly due to heightened immune cell infiltration. Recent findings identify chemerin as a key secretory protein driving an autocrine and paracrine network that promotes the mesenchymal phenotype of GBM cells [38]. Chemerin enhances these features by stabilizing its receptor, CMKLR1—predominantly expressed on TAMs—by preventing its ubiquitin-proteasomal degradation and upregulating the NF-kB pathway. Additionally, chemerin facilitates TAM recruitment and promotes immunosuppressive phenotype polarization via the CMKLR1/NF-kB axis, further reinforcing mesenchymal phenotype. Disruption of this chemerin/CMKLR1 signaling pathway via 2-(a-naphthoyl) ethyltrimethylammonium iodide effectively dismantled the mesenchymal network and reduced the infiltration of TAMs, leading to suppressed tumor growth in vivo [38].

Delivery of R848, a potent dual TLR7/8 agonist, via cyclodextrin nanoparticles (CDNP) has gained recent attention as a novel therapeutic strategy to deplete TAM infiltration [39]. In one study, CDNP-R848 treatment significantly reshaped the TME by enhancing the pro-inflammatory activity of myeloid cells and promoted tumor cell death independent of T and NK cells. The treatment also significantly reduced the recruitment of immunosuppressive macrophages to the tumor site and improved survival in the syngeneic mouse model [39]. Cytokine profiling revealed elevated IL-12 and other pro-inflammatory cytokines that indicate successful activation of the Toll-Like Receptor 7/8 (TLR7/8) pathway. Importantly, CDNP-R848 reduced immunosuppressive Tregs and decreased Lymphocyte-Activation Gene 3 (LAG-3) expression, further favoring a pro-inflammatory TME. Imaging techniques confirmed reduced macrophage recruitment, highlighting the potential of CDNP-R848 as an effective strategy to reduce recruitment and improve the pro-inflammatory, anti-tumor response [39]. 

### 5.2. Selective Depletion Within the TME

The p38/MAPK pathway promotes the recruitment of macrophages and microglia while also increasing PD-L1 expression in tumor cells and TAMs. Inhibiting this pathway, combined with anti-PD-L1 antibody therapy, reduces macrophage infiltration and lowers PD-L1 expression in microglia, enhancing survival in TMZ-resistant GBM models. This combination also decreases circulating monocyte-derived TAMs and PD-L1 in resident microglia, suggesting a potential therapeutic approach for recurrent or chronic TMZ-resistant GBM [40]. 

Additional molecular targets under investigation include the Phosphoinositide 3-kinase-γ (PI3Kγ) pathway which plays a crucial role in promoting microglia chemotaxis and IL-11 secretion [27]. Thus, inhibition of this pathway disrupts the signaling and recruitment of immunosuppressive myeloid-derived cells and downstream activation of the Signal Transducer and Activator of Transcription 3 (STAT3) pathway leads to tumor cell proliferation. In addition, PI3Kγ inhibition synergistically enhanced the effects of temozolomide (TMZ) in preclinical studies [27]. A tangent of the PI3Kγ pathway is the mammalian target of rapamycin (mTOR) signaling pathway that has demonstrated effects of modulating the TME. mTOR belongs to the PI3K-related family and plays a role in growth and proliferation of GBM cells [41]. Using a blood–brain barrier-penetrating liposomal system for CDX-modified liposomes (termed CDX-LIPO, and the non-modified liposomes termed LIPO), negative regulation of the mTOR pathway led to remarkable tumor cell death and simultaneous stimulation and activity of pro-tumor macrophages and T cells [41]. 

Ponatinib, a multi-targeted receptor tyrosine kinase inhibitor, has previously demonstrated efficacy in binding to PD-L1 and inhibiting melanoma growth, so one study assessed its ability to induce antitumor immunity in a murine GBM model [42]. Results indicated that Ponatinib was able to reduce PD-L1 expression, increase CD8^+^ T cell infiltration, and modulate the ratio of immunosuppressive to pro-inflammatory TAMs by depleting immunosuppressive macrophages. In addition, forkhead box P3 (FOXP3) expression was significantly reduced leading to a decrease in Tregs. All of this equated to a shift away from an immunosuppressive TME with increased T cell activation and pro-inflammatory response [42]. 

## 6. Reprogramming Macrophages

**Figure 3:**
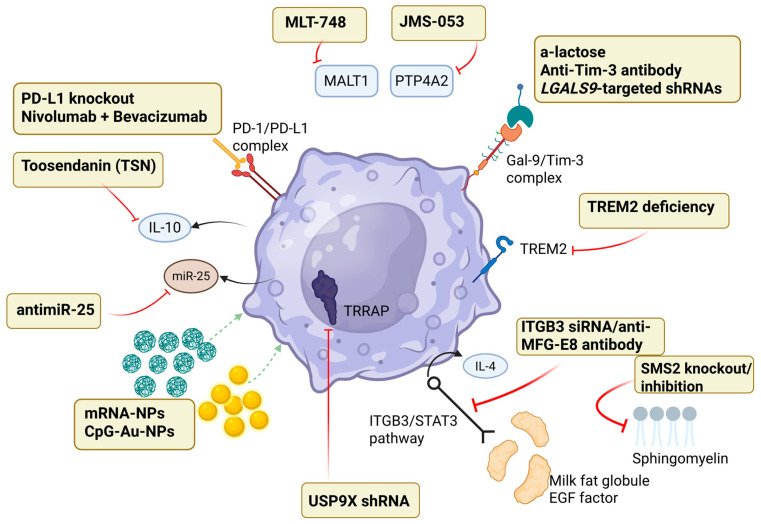
Therapeutic targets to reprogram macrophages to a pro-inflammatory phenotype include PD-1/PD-L1 blockade, MALT1 inhibition, Gal-9/Tim-3 blockade, IL-10 suppression, miR-25 extracellular vesicles, ITGB3/STAT3 pathway inhibition, sphingomyelin reduction, PTP4A2 reduction, and TRRAP inhibition. Nanoparticles (mRNA-NPs, CpG-Au-NPs) are also employed to module the TAM immune response.

Macrophages exhibit considerable functional plasticity, allowing dynamic shifts between immunosuppressive and pro-inflammatory phenotypes within the tumor microenvironment (TME) [43]. Redirecting immunosuppressive tumor-associated macrophages (TAMs) towards pro-inflammatory, anti-tumor phenotypes presents an attractive therapeutic strategy for enhancing tumor suppression (Figure 3) [43].

### 6.1. Immune Checkpoints

Tumor cells commonly evade immune detection by upregulating immune checkpoint molecules such as programmed death-1 (PD-1) and its ligand PD-L1. PD-1 is notably expressed not just by tumor cells but also by immunosuppressive TAMs and has become a target for repolarization. Elevated PD-1 expression in TAMs correlates with reduced phagocytic activity and weakened anti-tumor responses. In colorectal cancer, increased PD-1 levels in macrophages correlate with larger tumors and more advanced stages, especially within immunosuppressive subsets [44]. Blocking PD-1/PD-L1 pathways can restore macrophage function, enhancing phagocytosis and limiting tumor growth [45]. In glioblastoma (GBM), using dual-sgRNA CRISPR/Cas9 technology to remove both surface and intracellular PD-L1 from human U87 GBM cells resulted in a 64% PD-L1 reduction, significantly decreased cell migration, and promoted pro-inflammatory macrophage polarization. These TAMs showed elevated TNF-α and reduced IL-4 secretion, contributing to stronger anti-tumor immunity [46].

### 6.2. Intracellular Signaling Pathways

GBM frequently has phosphatase and tensin homolog (PTEN) deficiencies, increasing the secretion of lysyl oxidase (LOX) and galectin-9 (Gal-9). LOX attracts macrophages, while Gal-9 suppresses immune activity by interacting with T-cell immunoglobulin and mucin domain 3 (Tim-3) [47]. Inhibiting Gal-9/Tim-3 signaling, either with anti-Tim-3 antibodies or genetic knockdown methods, effectively reduces immunosuppressive TAM polarization and slows tumor proliferation in PTEN-null GBM [48]. Furthermore, Galectin-3 (Gal-3) depletion increases inducible nitric oxide synthase (iNOS) expression and lowers arginase-1 (Arg-1), favoring pro-inflammatory polarization. Temozolomide (TMZ) enhances this pro-inflammatory shift in Gal-3-deficient mice, as confirmed by increased pro-inflammatory gene expression [49]. 

Milk fat globule EGF factor-8 (MFG-E8) contributes to immunosuppression in GBM by activating the integrin β3 (ITGB3)/STAT3 pathway. This signaling promotes IL-4 secretion, driving microglia toward immunosuppressive states with markers such as arginase-1 (ARG-1), macrophage galactose-type C-type lectin-2 (MGL-2), and the macrophage mannose receptor (CD206) [46,50]. Recombinant MFG-E8 amplifies IL-4-driven immunosuppressive polarization while suppressing pro-inflammatory responses induced by lipopolysaccharide (LPS). Blocking MFG-E8 or integrin β3 promotes microglia pro-inflammatory polarization, reduces ARG-1 expression, and limits tumor growth. MFG-E8 knockdown in glioma cells also boosts chemotherapy efficacy (BCNU), reduces tumor size, prolongs survival, and decreases immunosuppressive macrophage infiltration [50]. Thus, MFG-E8 inhibition offers a valuable approach to reprogramming macrophages and combating GBM-induced immunosuppression. 

Recent research identifies MALT1 protease as a key regulator of TAM immunosuppressive polarization in GBM. Elevated MALT1 expression is found in aggressive mesenchymal GBM macrophages. Using the MALT1 inhibitor MLT-748 reverses immunosuppressive polarization, boosts macrophage-mediated tumor destruction, and extends survival in GBM animal models. MALT1 knockout mice exhibit increased pro-inflammatory macrophage populations compared to wild-type mice [51]. 

The small-molecule compound Toosendanin (TSN) has demonstrated potential in reinforcing macrophage-mediated anti-tumor responses in GBM. TSN inhibits IL-10, substantially reducing immunosuppressive macrophage markers and promoting pro-inflammatory cytokines like IL-1β and IL-8 [52]. In animal models, TSN reduced tumor size, increased CD8^+^ T-cell infiltration, reduced regulatory T cells (Tregs), and decreased T-cell exhaustion. Combining TSN with checkpoint inhibitors or EGFRvIII CAR-T therapy provided even better tumor control, longer survival, and enhanced T-cell activation [52]. 

### 6.3. Nanotechnology and Delivery Systems

Targeted therapeutic delivery to TAMs, exploiting macrophage phagocytic behavior, has become an innovative method to modify macrophage polarization. Nanoparticles carrying mRNA encoding interferon regulatory factor 5 (IRF5) and activating kinase IKKβ successfully shifted TAM polarization to anti-tumor states [53]. While these nanoparticles alone modestly improved survival, combining them with radiotherapy substantially reduced tumor growth and significantly extended survival in preclinical studies [53]. Similarly, gold nanoparticles loaded with cytosine–phosphate–guanosine (CpG), a toll-like receptor 9 (TLR-9) agonist, effectively increased pro-inflammatory cytokine production by macrophages and activated CD8^+^ T cells, potentially altering GBM’s immunosuppressive environment [54].

GBM promotes immunosuppressive TAM polarization via extracellular vesicles containing microRNA-25 (miR-25), which suppresses the cyclic GMP-AMP synthase (cGAS)-stimulator of interferon genes (STING) pathway. Combining antimiR-25 with cyclic GMP-AMP (cGAMP) nanoparticles activates cGAS-STING, shifting macrophages toward pro-inflammatory phenotypes and enhancing M1-associated genes, including Cxcl10 and Ifna2 [55]. 

### 6.4. Emerging Targets

Phosphatase of regenerating liver 2 (PRL2), encoded by PTP4A2, and ubiquitin-specific protease 9X (USP9X) also modulate TAM polarization. Increased PRL2 is associated with aggressive GBM and poorer outcomes. The PRL inhibitor JMS-053 reduces GBM cell viability, induces apoptosis, and modulates macrophages toward pro-inflammatory states [56]. Conversely, USP9X stabilizes TRRAP protein, enhancing GBM cell proliferation and migration. USP9X knockdown reduces tumor growth, lowers TRRAP expression, and shifts macrophages toward pro-inflammatory states by decreasing IL-10 and TGF-β1 secretion [57]. 

Triggering receptors expressed on myeloid cells 2 (TREM2) significantly influences TAM polarization and immune suppression in GBM. Increased TREM2 levels predict poorer outcomes [58]. Genetic deletion of TREM2 in human and mouse myeloid cells enhances interferon-gamma-induced activation, promotes pro-inflammatory polarization, and increases anti-tumor activity. TREM2 inhibition reduces tumor growth and extends survival by increasing infiltration of PD-1-positive cytotoxic T lymphocytes and reducing immunosuppressive macrophage numbers [58]. TREM2 blockade also boosts anti-PD-1 checkpoint inhibitor efficacy. 

Recent studies have highlighted the pivotal role of lipid metabolism in modulating TAM function. Alterations in lipid pathways—including fatty acid oxidation, cholesterol homeostasis, and sphingolipid metabolism—have been shown to influence macrophage polarization [59,60]. Therapeutic strategies targeting key enzymes such as fatty acid synthase (FASN) and acetyl-CoA carboxylase (ACC) reduce lipid accumulation in TAMs, leading to decreased production of immunosuppressive cytokines and enhanced antigen presentation [61,62]. Moreover, modulation of cholesterol metabolism has been associated with improved phagocytic activity and a shift toward a pro-inflammatory phenotype in TAMs [60,63]. These findings underscore the potential of lipid manipulation as an innovative approach to reprogram immunosuppressive macrophages in GBM. 

Lastly, complement factors (C1q, C3a/C3aR, C5a/C5aR) and enzymes regulating sphingomyelin metabolism (SMS2, SMPD1) significantly influence macrophage polarization. Targeting these molecules can modulate immune responses, encourage anti-tumor macrophage polarization, and restrict GBM progression, presenting additional promising avenues for GBM immune modulation therapies [64,65,66,67,68]. 

## 7. Targeting Downstream Pro-Tumor Signals

**Figure 4:**
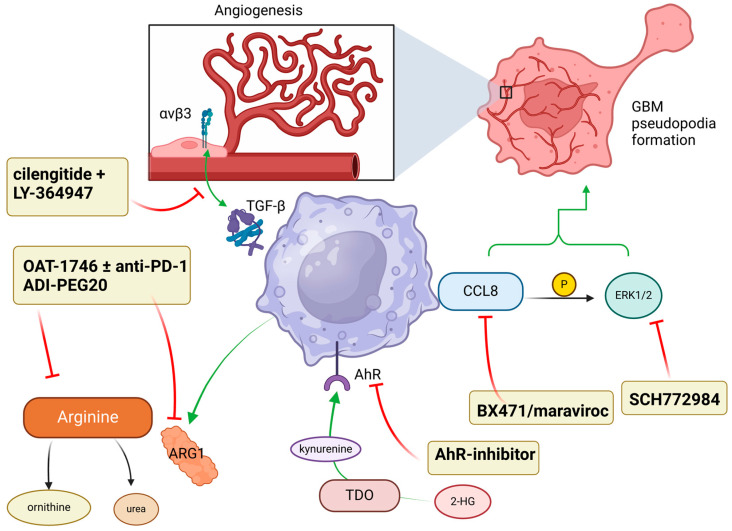
Therapeutic downstream TAM targets to reverse immunosuppression and shift towards a pro-inflammatory TME. Angiogenesis via integrin αvβ3-TGF-β signaling, GBM pseudopodia formation from CCL8 and ERK1/2 phosphorylation, arginine metabolism, and kynurenine-AhR interaction are potential pathways.

Macrophage-driven arginine metabolism and downstream signaling cascades play a crucial role in orchestrating immunosuppression, making these pathways critical targets for intervention (Figure 4).

Arginine metabolism significantly influences immune responses in the tumor microenvironment (TME), primarily through its modulation by macrophages, thus impacting tumor progression [69]. Pro-inflammatory macrophages metabolize arginine through inducible nitric oxide synthase (iNOS) to produce nitric oxide (NO), a molecule that can efficiently eliminate tumor cells. In contrast, immunosuppressive macrophages utilize arginase enzymes (ARG1 and ARG2) to convert arginine into ornithine and urea, leading to local immune suppression [70]. Both human and mouse glioblastomas (GBMs) demonstrate elevated expression of ARG1 and ARG2 in tumor cells and infiltrating macrophages and microglia, causing a depletion of L-arginine, which is essential for activating T cells and natural killer (NK) cells [71]. Targeting this pathway, the selective arginase inhibitor OAT-1746 effectively reduced glioma cell invasion mediated by microglia, restored arginine levels in the brain, and decreased tumor growth. Additionally, combining OAT-1746 with PD-1 blockade further enhanced therapeutic effects, increasing the infiltration of CD8^+^ T cells. These findings illustrate that the inhibition of ARG1/2 could augment immune checkpoint blockade efficacy, restore immune functionality, and boost anti-tumor responses [71]. 

Moreover, combining radiotherapy with the arginine-depleting agent ADI-PEG20 has proven highly effective in an orthotopic GBM model, achieving substantial tumor regression and extending disease-free survival beyond 140 days. ADI-PEG20 treatment not only increased radiosensitivity of tumor cells but also reshaped the macrophage and microglia populations, driving their polarization towards a phagocytic and pro-inflammatory state [72]. This immune shift was indicated by increased intracellular lipid accumulation and elevated nitric oxide levels. Transcriptomic analysis further confirmed a decrease in the immunosuppressive marker Arg-1 and an increase in the pro-inflammatory cytokine TNF-α. Thus, combining arginine depletion and radiotherapy effectively enhances anti-tumor immunity [72]. 

Chemokine (C-C motif) ligand 8 (CCL8), abundantly produced by tumor-associated macrophages (TAMs), significantly promotes the invasiveness of GBM cells by stimulating pseudopodia formation [73]. Three-dimensional culture experiments demonstrated that exposure to CCL8 notably increased cell migration and invasive potential in GBM cell lines. Further analysis using immunoblotting revealed the enhanced phosphorylation of extracellular signal-regulated kinase (ERK1/2) upon CCL8 stimulation. Treatment with BX471 and maraviroc, inhibitors of CCL8 signaling, diminished ERK1/2 activation [73]. Furthermore, the ERK1/2-specific inhibitor SCH772984 markedly reduced CCL8-induced invasive characteristics and pseudopodia formation. Neutralizing CCL8 using an anti-CCL8 antibody significantly decreased GBM cell migration and ERK1/2 activation triggered by TAM-secreted factors. These findings suggest that the CCL8-ERK1/2 signaling pathway represents a valuable target for limiting GBM invasiveness [73]. 

Immunosuppressive macrophages further contribute to tumor progression by promoting angiogenesis through direct interactions between transforming growth factor-beta 1 (TGF-β1) and integrin αvβ3, highly expressed on angiogenic endothelial cells [74]. Pro-tumor macrophages engage integrin αvβ3, stimulating new blood vessel growth. Combined therapy using cilengitide, an αvβ3 integrin antagonist, and LY-364947, a TGF-β1 receptor inhibitor, demonstrated superior efficacy in reducing angiogenesis compared to the VEGF inhibitor cediranib alone. This suggests that dual inhibition of this pathway could significantly suppress GBM angiogenesis [74]. 

Gliomas harboring isocitrate dehydrogenase 1/2 (IDH1/2) mutations exhibit the CpG island methylator phenotype (CIMP) and are typically associated with improved survival compared to their wild-type counterparts [75]. Nevertheless, IDH-mutant gliomas accumulate significant amounts of 2-hydroxyglutarate (2-HG), contributing to profound immune suppression with notably decreased infiltration of macrophages, dendritic cells, and lymphocytes [75]. The accumulation of 2-HG suppresses immunity by downregulating critical chemokines such as CXCL10, hindering T-cell maturation, and promoting immunosuppressive polarization of macrophages via the activation of tryptophan-2,3-dioxygenase (TDO) [76]. Activation of TDO increases kynurenine production, stimulating the aryl hydrocarbon receptor (AhR), which further reinforces immunosuppressive polarization in macrophages [76]. Importantly, the inhibition of AhR successfully restores a pro-inflammatory, anti-tumor macrophage phenotype in IDH-mutant gliomas, and combining AhR inhibitors with anti-PD-L1 immunotherapy synergistically enhances therapeutic outcomes [76]. 

Insights into macrophage plasticity within the GBM microenvironment provide a foundation for translating preclinical strategies into clinical application. Approaches such as macrophage depletion, reprogramming toward pro-inflammatory phenotypes, and targeting immunosuppressive signaling pathways offer promising directions (Table 1). These strategies’ feasibility and therapeutic potential are being explored in human trials. 

## 8. In Human Trials

Recent clinical trials have begun translating preclinical discoveries into early-phase studies evaluating the safety, feasibility, and immunomodulatory impact of TAM-directed therapies (Table 2). Translating preclinical insights into clinical application, a phase I/II study examined the safety and efficacy of Macrophage Exclusion after Radiation Therapy (MERT) using plerixafor, a CXCR4 inhibitor, in newly diagnosed GBM (ndGBM) patients [77]. The rationale for this trial stemmed from evidence indicating that radioresistant hypoxic cells and hypoxia-inducible factor-1 (HIF-1) upregulation elevate stroma cell-derived factor-1 (SDF-1), which recruits bone marrow-derived monocytes through CXCR4 receptors, promoting angiogenesis and tumor recurrence. Preclinical animal models demonstrated that disrupting the SDF-1/CXCR4 axis limited macrophage infiltration, improved radiotherapy outcomes, and increased survival. Patients received plerixafor via continuous IV infusion for four weeks post-standard radiochemotherapy [77]. The treatment showed favorable safety, with no grade 3 toxicities, and clinical outcomes included a median overall survival (mOS) of 21.3 months and progression-free survival (PFS) of 14.5 months. Biomarker analyses indicated a successful CXCR4 blockade, increased SDF-1 levels, and a reduction in cerebral blood volume within irradiated regions, suggesting reduced microvascular proliferation. Thus, CXCR4 inhibition, in conjunction with standard therapy, demonstrated promising potential for improved tumor control [77]. 

Building on this, a subsequent phase I study evaluated the combination of plerixafor with bevacizumab in recurrent GBM (rGBM) patients to determine safety and preliminary efficacy [85]. Conducted in two cohorts—a dose-escalation phase with 23 patients and a surgical phase with 3 patients—the study reported good tolerability and no dose-limiting toxicities. Pharmacokinetic data confirmed effective plerixafor penetration into cerebrospinal fluid and tumor tissue, achieving therapeutic CXCR4-inhibiting concentrations. Biomarker assessments showed elevated plasma SDF-1 and reduced free VEGF levels, aligning with effective inhibition of both CXCR4 and VEGF pathways. Despite these promising pharmacodynamic results, clinical outcomes were modest, yielding a mOS of 7.1 months and median PFS (mPFS) of 2.87 months [85].

Extending macrophage-targeted strategies, a phase I trial combined immunoadjuvants with radiotherapy in rGBM patients, exploring the immunological effects and clinical outcomes of polyinosinic/polycytidylic acid (poly I/C) and granulocyte-macrophage colony-stimulating factor (GM-CSF) [82]. Poly I/C activates toll-like receptor-3 (TLR3) to induce pro-inflammatory cytokines, while GM-CSF facilitates macrophage differentiation and activation. Among 30 patients treated, 20% exhibited objective responses, with an mPFS of 88 days and mOS of 362 days. Notably, responders showed increased infiltration of CD8^+^ T lymphocytes and NK cells, whereas non-responders displayed elevated CD68+ macrophages, suggesting persistent immunosuppressive macrophage presence correlated with poorer outcomes [82]. 

Focusing on macrophage modulation via colony-stimulating factor 1 receptor (CSF1-R) inhibition, PLX3397—an oral, brain-penetrant small-molecule inhibitor—was evaluated in a phase II clinical trial for patients with recurrent GBM [79]. Recognizing the significant expression of CSF1-R ligands in GBM, the trial enrolled 37 patients divided into surgical (n = 13) and non-surgical (n = 24) cohorts. Although PLX3397 was well tolerated and reached therapeutic concentrations in tumor tissues, the primary endpoint—6-month PFS—was modest at 8.8%. Genomic analysis failed to identify biomarkers predictive of extended responses, highlighting the complexity of targeting CSF1-R pathways in GBM treatment [79]. 

Another innovative approach involved D2C7 immunotoxin (D2C7-IT), a recombinant protein targeting wild-type and mutant EGFR (EGFRvIII), conjugated with Pseudomonas exotoxin for direct tumor cell killing and immune activation [83]. D2C7-IT induces T cell activation and upregulates inflammatory markers such as CD40, creating synergy when combined with anti-CD40 agonist antibodies like 2141-V11. A first-in-human trial currently evaluates the safety and maximum tolerated dose of intratumorally administered D2C7-IT combined with escalating doses of 2141-V11 in rGBM patients. Preliminary results from eight patients indicated favorable tolerability, with minimal adverse events including headaches and transient neurological symptoms [83].

Finally, exploring strategies targeting macrophage inhibitory factor (MIF), a phase 1b/2a dose-escalation study investigated ibudilast—a selective MIF inhibitor—in combination with temozolomide (TMZ) for both newly diagnosed and recurrent GBM patients [84]. By disrupting the MIF-CD74 interaction, ibudilast reduces immunosuppressive properties of myeloid-derived suppressor cells (MDSCs), which, under tumor-induced conditions, preferentially differentiate into immunosuppressive macrophages and Tregs. In this clinical trial, 36 newly diagnosed and 26 recurrent GBM patients were enrolled, receiving monthly TMZ with daily ibudilast doses escalating from 30 mg to 50 mg twice daily. Outcomes included a 6-month PFS rate of 44% for newly diagnosed GBM and 31% for recurrent GBM, with a mOS of 21.0 months and 8.6 months, respectively, supporting the feasibility of MIF inhibition to modulate the immunosuppressive microenvironment [84]. 

## 9. Discussion

Despite the minimal success of T cell-based therapies for glioblastoma (GBM), the role of tumor-associated macrophages (TAMs) and TAM-associated pathways remains underexplored. GBM remains a significant therapeutic challenge, characterized by an immunosuppressive tumor microenvironment (TME) that severely limits the efficacy of conventional treatments and adaptive immune-targeting therapies. TAMs have emerged as central regulators of this immunosuppression due to their considerable presence, plasticity, and their ability to dynamically shift along a polarization continuum from pro-inflammatory (anti-tumor) to immunosuppressive (pro-tumor) phenotypes. 

Recent advancements utilizing transcriptomic and proteomic technologies have revealed TAM complexity and plasticity, highlighting opportunities to therapeutically target macrophage phenotypic reprogramming. Preclinical studies targeting macrophages through depletion, polarization modulation, and downstream signaling disruption have demonstrated promising potential to enhance antitumor responses and reduce tumor growth. Strategies such as CSF-1R inhibition, the blockade of chemokine axes (CCL2/CCR2), interference with immunosuppressive signaling pathways (adenosine/CD73, PI3Kγ, mTOR, and MAPK pathways), and nanoparticle-mediated delivery of macrophage-modulating agents have produced encouraging preclinical outcomes. 

Clinical translation of these macrophage-focused strategies has started, with early-phase human trials investigating TAM depletion or reprogramming using agents such as plerixafor, CSF-1R inhibitors (PLX3397), immunotoxins (D2C7-IT), and MIF inhibition (ibudilast). Although early results underscore the safety and feasibility of these approaches, clinical efficacy remains modest, reinforcing the complexity of GBM-associated immunosuppression and highlighting the need for integrated, multimodal strategies. 

This review uniquely consolidates the emerging evidence on TAM-targeted strategies and promotes its use in targeting the immunosuppressive TME in GBM. In contrast to the previous literature that often presents TAMs within a binary M1/M2 paradigm, this work emphasizes their dynamic polarization along a continuum and highlights their functional diversity. The next phase of immunotherapy research in GBM should focus on the design of multimodal, macrophage-centric immunotherapies aimed at overcoming the tumor’s immunosuppression. In particular, therapies targeting TAM polarization can greatly influence the TME by transforming it into a pro-inflammatory phenotype with enhanced T-cell infiltration and activation and reduced immunosuppressive signaling.

## 10. Conclusions

Moving forward, effective therapeutic approaches must leverage a nuanced understanding of TAM biology, including molecular subtypes, macrophage functional plasticity, and interplay with other immune and stromal cells within the TME. The integration of macrophage-targeted therapies with established and emerging treatment modalities—such as checkpoint inhibitors, radiotherapy, and advanced immunotherapeutics—holds promise for overcoming current barriers and significantly improving clinical outcomes in GBM patients. 

## Figures and Tables

**Table 1:** Immunotherapeutic strategies targeting TAMs and associated pathways in GBM.

Strategy	Treatment	Mechanism	TAM Effect
Depletion	TG100-115 [27]	PI3K γ inhibition	Suppresses microglia/TAM accumulation and secretion of IL-11
	BLZ945 + OSI906 [32]	CSF-1R + IGF-1R inhibition	Prevent immunosuppressive TAM trafficking
	PLX3397 + cediranib [33]	CSF-1R + VEGFR2 inhibition	Prevent immunosuppressive TAM trafficking and reduce angiogenesis
	NE-siRNA-CD73 [34]	CD73 inhibition	Reduce Tregs, microglia, and macrophages
	CCX872/Anti-PD1 [35]	CCL2/CCR2 axis disruption	Reduce tumor-associated MDSCs and T lymphocyte exhaustion
	CCR2 knockout [36]	CCL2/CCR2 axis disruption	Decrease TAM infiltration
	Celecoxib [37]	CCL2 and CXCL10 inhibition	Reduce microglia and macrophages
	2-(a-naphthoyl) ethyltrimethylammonium iodide [38]	Chemerin/CMKLR1 pathway disruption	Reduce TAM infiltration
	R848 via CDNP [49]	TLR7/8 agonism	Increase pro-inflammatory cytokines and reduce immunosuppressive TAM infiltration
	LY2228820 [40]	p38 MAPK inhibitor	Inhibit macrophage aggregation
	CDX-LIPO [41]	Target PI3K/mTOR	Promote pro-inflammatory phenotype and deplete immunosuppressive TAMs
	Ponatinib [42]	Inhibit PD-L1	Deplete immunosuppressive TAMs
Reprogramming	Nivolumab/Bevacizumab [44]	Inhibit PD-L1 pathway	Regulate TAM polarization
	PD-L1 knockout [45]	Inhibit PD-L1 pathway via dual-sgRNA CRISPR/Cas9	Upregulate pro-inflammatory phenotype, downregulate immunosuppressive phenotype
	a-lactose [47]	Gal-9/Tim-3 blockade	Inhibit immunosuppressive polarization and VEGF release
	Anti-Tim-3 antibody [48]	Gal-9/Tim-3 blockade	Inhibit immunosuppressive phenotype
	*LGALS9*-targeted shRNAs [48]	Gal-9/Tim-3 blockade	Inhibit immunosuppressive phenotype
	ITGB3 siRNA/anti-MFG-E8 antibody [50]	MFG-E8 pathway	Increase M1-like microglia, decrease M2-like microglia
	MLT-748 [51]	MALT1 inhibition	Reverses immunosuppressive polarization
	Toosendanin (TSN) [52]	IL-10 inhibition	Reduce immunosuppressive TAM markers and promote pro-inflammatory cytokines
	mRNA-NPs [53]	IRF5/IKKb expression	Upregulate pro-inflammatory phenotype, downregulate immunosuppressive phenotype
	CpG-Au-NPs [54]	Enhance immunosuppressive macrophage repolarization	Upregulate pro-inflammatory phenotype, downregulate immunosuppressive phenotype
	microRNA-25 antibody (antimiR-25) [55]	STING pathway suppression	Promotes pro-inflammatory phenotype polarization
	JMS-053 [56]	PRL inhibition	Induces apoptosis and promotes pro-inflammatory polarization
	USP9X knockout [57]	De-stabilizes TRRAP protein	Lowers TRRAP expression and shifts TAMs towards pro-inflammatory phenotype
	TREM2 knockout [58]	Decreased TREM2 activation	Promotes pro-inflammatory polarization
	SMS2 knockout [67]	Inhibition of sphingomyelin production	Suppress immunosuppressive TAM polarization
Targeting downstream signals	OAT-1746 [71]	Inhibiting ARG1/2	Polarize TAM to pro-pro-inflammatory phenotype
	ADI-PEG20 [72]	Arginine depletion	Promote pro-inflammatory polarization and increase radiosensitivity of GBM cells
	BX471 and maraviroc [73]	CCL8-antibody and ERK1/2 inhibition	Decrease macrophage-derived CCL8
	SCH772984 [73]	ERK1/2 inhibition	Reduce macrophage-derived CCL8 and decrease pseudopodia formation
	Cilengitide + LY-364947 [74]	Inhibition of integrin αvβ3 and TGF-β, respectively	Inhibit endothelial cells-macrophage interaction and reduce angiogenesis
	AhR-inhibitor [76]	Inhibit AhR-kynurenine interaction	Reverse immunosuppression

**Table 2:** Clinical trials targeting TAM-associated pathways and functions in patients with glioblastoma, accessed on 7 March 2025.

ID	Author	Phase	Mechanism	TAM Effect	Patient Population	Outcomes	Recruitment Status
NCT01977677 [77]	Thomas et al.	I/II	Inhibition of SDF-1/CXC4 axis	Prevent TAM recruitment	ndGBM	mOS 21.3 months, PFS 14.5 months	Completed
NCT03746080 [78]	Recht et al.	II	Inhibition of SDF-1/CXC4 axis + WBRT + SOC	Prevent TAM recruitment	ndGBM	6-month PFS rate 91.7%, mOS 15.1 months	Active, not recruiting
NCT01349036 [79]	Butowski et al.	II	PLX3397 (CSF1-R inhibition)	Deplete immunosuppressive TAMs	rGBM	6-month PFS rate 8.8%	Terminated
NCT01790503 [80]	Sankyo et al.	I/II	PLX3397 + SOC	Deplete immunosuppressive TAMs	ndGBM	mPFS 6.7 months	Completed
NCT02829723 [81]	Novartis	I/II	BLZ945 (CSF1-R inhibition)	Deplete immunosuppressive TAMs	rGBM	6-month PFS rate 15.2%	Terminated
NCT03392545 [82]	Jiang et al.	I	GM-CSF + poly I/C + radiation	Reprogramming TAM polarization	rGBM	No results posted	Unknown
NCT04547777 [83]	Desjardins et al.	I	Anti-CD40 + D2C7-IT	Reprogramming TAM polarization	rGBM	No results posted	Recruiting
NCT03782415 [84]	Youssef et al.	I/II	Inhibition of MIF	Prevent MDSC differentiation into immunosuppressive TAM	ndGBM and rGBM	mOS 21.0 (ndGBM) and 8.6 (rGBM)	Active, not recruiting
